# The Insulation of Electric Light Wires

**Published:** 1881-06

**Authors:** 


### THE INSULATION OF ELECTRIC LIGHT WIRES.

At a meeting of the New York Board of Fire Insurance Underwriters, the danger arising from the use of electric lights with uninsulated conductors came up for discussion. The matter had been investigated on account of an accident a short time ago in a jewelry store in Maiden Lane, when a man was on the roof running an electric light wire across. It came in contact with the telephone wire, and a flash passed down to the telephone box, destroying it. The shock loosened a considerable extent of plaster.

City Electrician Smith said that the shock must, he thought, have been very powerful, and had any one been at the telephone, he might have been killed ; or if the flame had passed near light goods, there might have been a conflagration. The wire of the electric light ought to be thoroughly insulated.

Superintendent Harrison, of the New York Board of Fire Insurance Underwriters, said that the Board would ask the proper authorities to see that the electric wires were properly insulated. Owing to the rapid introduction of the electric light, and the many new wires that were being run over the city houses, the danger, he said, was constantly increasing. In the meantime buildings using the electric light would be rated as "specially hazardous," unless the insulation of the wire was approved.

A. A. Hayes, Jr., of the Brush Electric Lighting Company, has informed the board that the wires of that company were already insulated while the matter was under discussion ; and since the action of the Board, the other companies have been experimenting in regard to the best method of insulation.—*Scientific American.*

